# Evaluation of Mechanical and Electrical Performance of Aging Resistance ZTA Composites Reinforced with Graphene Oxide Consolidated by SPS

**DOI:** 10.3390/ma15072419

**Published:** 2022-03-25

**Authors:** Sergey Grigoriev, Anton Smirnov, Nestor Washington Solis Pinargote, Oleg Yanushevich, Natella Kriheli, Olga Kramar, Yuri Pristinskiy, Pavel Peretyagin

**Affiliations:** 1 Spark Plasma Sintering Research Laboratory, Department of High-Efficiency Machining Technologies, Moscow State University of Technology “STANKIN”, Vadkovsky per. 1, Moscow 127055, Russia; s.grigoriev@stankin.ru (S.G.); nw.solis@stankin.ru (N.W.S.P.); y.pristinskiy@stankin.ru (Y.P.); 2 Scientific Department, A.I. Yevdokimov Moscow State University of Medicine and Dentistry, Moscow 127473, Russia; Olegyanushevich@mail.ru (O.Y.); nataly0088@mail.ru (N.K.); dr.ovkramar@gmail.com (O.K.)

**Keywords:** graphene, graphene oxide, alumina, zirconia, spark plasma sintering, microstructure, mechanical properties, low-temperature degradation

## Abstract

This paper presents a study of Al_2_O_3_–ZrO_2_ (ZTA) nanocomposites with different contents of reduced graphene oxide (rGO). The influence of the rGO content on the physico-mechanical properties of the oxide composite was revealed. Graphene oxide was obtained using a modified Hummers method. Well-dispersed ZTA-GO nanopowders were produced using the colloidal processing method. Using spark plasma sintering technology (SPS), theoretically dense composites were obtained, which also reduced GO during SPS. The microstructure, phase composition, and physico-mechanical properties of the sintered composites were studied. The sintered ZTA composite with an in situ reduced graphene content of 0.28 wt.% after the characterization showed improved mechanical properties: bending strength was 876 ± 43 MPa, fracture toughness—6.8 ± 0.3 MPa·m^1/2^ and hardness—17.6 ± 0.3 GPa. Microstructure studies showed a uniform zirconia distribution in the ZTA ceramics. The study of the electrical conductivity of reduced graphene oxide-containing composites showed electrical conductivity above the percolation threshold with a small content of graphene oxide (0.28 wt.%). This electrical conductivity makes it possible to produce sintered ceramics by electrical discharge machining (EDM), which significantly reduces the cost of manufacturing complex-shaped products. Besides improved mechanical properties and EDM machinability, 0.28 wt.% rGO composites demonstrated high resistance to hydrothermal degradation.

## 1. Introduction

Thanks to their elevated values of strength, hardness, wear and corrosion resistance, as well as biocompatibility, oxide ceramics based on aluminum oxide (Al_2_O_3_) and zirconium dioxide (ZrO_2_) are commonly used in a wide variety of applications in many industries [1,2,3]. Some information on the ceramics’ properties and applications is shown in Table 1.

These ceramics and composites are used to manufacture precision instruments, nozzles, PVD targets, optical devices, bearings, artificial jewelry, cases for elite watches, etc. [6,7]. In addition, alumina and zirconia are used as biomaterials mainly in dental and joint replacement applications. It is necessary to remember that the Al_2_O_3_ is brittle [8] despite the good properties of aluminum oxide. The crack resistance value is ~4 MPa·m^1/2^ and Al_2_O_3_ is sensitive to slow crack propagation when the stress intensity coefficient K_I_ is below the critical value K_IC_. The improvement of the mechanical properties of ZrO_2_, especially fracture toughness, is achieved through a polymorphic phase transformation known as phase transformation toughening [9]. Due to the presence of yttrium, magnesia, or other oxides, the metastable tetragonal high-temperature phase is stable at room temperature. When a crack propagates in such materials, the metastable phase transforms into a stable monoclinic polymorph with a corresponding increase in material volume by ~4–5%. However, in addition to its superior properties, Y-TZP has one critical disadvantage. Under “in vivo” conditions, it can spontaneously transform into the stable monoclinic form. This process is called low-temperature degradation (LTD) or aging [10] and increases the roughness of the implant surface, which contributes to increased wear and eventually leads to catastrophic implant failure [11,12,13,14,15]. In order to compensate for the low strength of alumina and the aging sensitivity of zirconia, a new group of ceramic materials containing Al_2_O_3_ and ZrO_2_ was proposed [16,17,18,19,20]. One such example is ceramic composites made of alumina-strengthened zirconia (ZTA). The design and development of these materials are considered promising since it is possible to combine the mechanical performance of Al_2_O_3_ and improve the crack resistance of composites due to the t → m transformation of ZrO_2_ without a significant lack of aging under the influence of body fluid. Pecharroman et al. reported that it is very important to control the level of ZrO_2_ in ZTA composites [21]. In order to avoid spontaneous transformation of ZrO_2_, this limit must be below the percolation threshold, which was found to be 16 vol.% or 22 wt.%. 

The mechanical properties of ceramic composites directly depend on the grain size and uniformity of phase dispersion. In this regard, spark plasma sintering was chosen as a method for the consolidation of powder materials. This method uses a pulsed direct current with simultaneous application of pressure on the material. Its main technical advantages are the high speed of heating and cooling, which makes it possible to reduce the processing time due to the simultaneous application of mechanical pressure and electrical impulses [22,23,24].

In addition to all of the above, the widespread use of ceramic products is limited by the complexity of their processing. Traditional methods using diamond tools are very expensive and energy-consuming. Therefore, alternative machining methods are needed. This could be electrical discharge machining (EDM). However, this method is only possible for materials with an electrical resistance below 100–300 Ω·cm [25,26,27]. Therefore, graphene oxide (GO) was added to the ceramic matrix to improve the electrical conductivity of ZTA. For the preparation of ceramic oxide slurries, GO is preferable to graphene because it can be homogeneously dispersed in water. Then, during spark plasma sintering under vacuum conditions, the graphene oxide is reduced to graphene. In addition to improving electrical conductivity, the presence of graphene also improves the mechanical properties of the ceramic matrix due to the unique structural characteristics of graphene and good bonding surfaces between graphene and matrix, as confirmed by previous studies [28,29,30,31,32,33,34,35].

The aim of this work was to prepare homogeneous mixtures of ceramic powders with the addition of GO by colloidal processing and freeze-drying to obtain alumina-based composites with 16 vol.% 3Y-TZP and various concentrations of GO and to study the microstructure, physico-mechanical properties, and aging resistance of these materials.

## 2. Materials and Methods

### 2.1. Graphene Oxide Preparation

A modified Hammers method was used to obtain graphene oxide (GO) from graphite powder. This process was described in more detail in previous papers [8,36,37]. In this work, commercial graphite powder (Plasmotherm, Moscow, Russia) with a median particle size d_50_ = 3 mm was used.

### 2.2. Powder Processing and Sintering

Commercial t-ZrO_2_ (3Y-TZP, 3 mol% Y_2_O_3_; TZ-3YS-E, Tosoh Corp., Tokyo, Japan), and alumina (α-Al_2_O_3_; A16SG, Alcoa, New York, NY, USA) powders with particle size d_50_ = 0.20 µm and d_50_ = 0.30 µm, respectively were used in this work. The content of the powder mixture was 84 vol.% of Al_2_O_3_ and 16 vol.% ZrO_2_. The necessary amount of powders was placed in a plastic container with Al_2_O_3_ balls (diameter 3 mm), distilled water, and Dolapix CE 64 as dispersant. Obtained mixture was wet mixed in a multi-directional mixer for 24 h at 150 rpm and subsequently dried by FreeZone2.5 freeze-drying system (LabConco, Kansas, MO, USA). Distilled water with the pH value of 10 was added to the produced mixture, which then was dispersed under mixing for 30 min. To obtain compositions with the required content of graphene oxide, it was added drop by drop to the suspensions and stirred for 1 h. To obtain the powder mixtures for further consolidation by SPS, resulting suspensions were dried at 110 °C in a Lab spray dryer (B-290, Buchi, Flawil, Switzerland). Thereby, ZTA powders with GO content of 0, 0.28 wt.% (0.5 vol%), 0.52 wt.% (1 vol.%), and 6.1 wt.% (5 vol.%) were prepared and labeled as 0-G, 0.5-G, 1-G, and 5-G, respectively. All powder mixtures were sintered in an H-HP D 25 SD Spark Plasma Sintering machine (FCT Systeme GmbH, Rauenstein, Germany) in vacuum at 1500 °C applying heating rate and pressure 100 °C/min and 80 MPa, respectively. The isothermal hold at final temperature was 3 min. The produced samples had diameter of 20 mm and a height of 3 mm. During sintering, the in situ thermal reduction of the GO takes place. In this work, the obtained carbon material after consolidation will be referred to as reduced graphene oxide (rGO).

### 2.3. Microstructural Characterization

A field emission scanning electron microscopy LYRA3 (Tescan, Brno, Czech Republic) equipped with an X-Act energy dispersive spectroscopy detector (Oxford Instruments, Abingdon, UK) was used to study the microstructure. Before the studies, the samples were polished using diamond polishing slurries with grit sizes ranging from 9 micrometers to one micrometer. After polishing, the samples were washed in ultrasound bath in ethanol for 15 min and dried using compressed air.

### 2.4. X-ray Diffraction (XRD), Raman Characterization

Phase identification of sintered samples and raw powders was carried out by X-ray Diffraction in an Empyrean diffractometer (PANalytical, Almelo, Netherlands) with radiation source Cu–Kα (λ = 1.5405981 Å) operated with an intensity of 30 mA at 40 kV [18] in the 2*θ* angle range of 5–70°. The analysis was carried out with a scanning speed of 0.06/min and a step size of 0.05. Raman analysis was achieved in a Raman analyzer DXR^TM2^, (Thermo Fisher Scientific, Waltham, MA, USA) using a 532 nm laser with a power of 2.0 mW for the control of the graphene-based mixtures and composites. A 50× optical microscope objective was applied to focus the laser beam on the studied area into a 2 μm spot with an accumulation time of about 10 s [28].

### 2.5. Aging Experiments

Aging experiments were performed at 134 °C for 30 h with a pressure of 200 kPa in an autoclave (Microclave 4001404, J.P. Selecta S.A., Barcelona, Spain). The t → m transformation control of ZrO_2_ was completed by X-ray diffraction on the sample surface by means of aging experiment interruptions at given times. The amount of m-ZrO_2_ and its volume fraction were estimated according to the Garvie and Nicholson [38] as well as the Toraya et al. [39] methods, respectively.

### 2.6. Mechanical Properties Characterization

The density of the composites was measured by Archimedes’ method in distilled water. The theoretical density was calculated using a ZrO_2_ density of 6.05 g/cm^3^, Al_2_O_3_ density of 3.98 g/cm^3^, and rGO density of 2.2 g/cm^3^.

Their Vickers hardness (*H_v_*) was measured from 10 footprints (indenter Qness, Salzburg, Austria) per sample under load and loading time 98 N and 10 s, respectively. To estimate the average of hardness values the following equation was used:

(1) $$H_{v}=\frac{0.1891\;P}{d^{2}}$$

where *P* was the set load (*N*); and *d* is the average length of two diagonals (mm).

The values of Vickers indentation fracture toughness, *K_C_*, were estimated by Equations (2) and (3) proposed by Miranzo and Moya [40].

(2) KC=0.047·P(d0.42c1.08)·[0.768·(EHv)0.05+0.612·Ln(EHv)−2]; Cd>2.8

(3) KC=0.0232·P(d·c1/2)·[0.768·(EHv)0.05+0.612·Ln(EHv)−2]; Cd<2.8

where *H_v_* is Vickers hardness of material (GPa), *P* is the applied load (N), *d* is the average length of the two diagonals (mm), *c* average length of cracks (mm), and *E* is the Young’s modulus of material (GPa).

The flexural strength (*σ_f_*) was evaluated through a biaxial bending test (ISO 6872). Each sample was placed onto a device with three balls of 3 mm in diameter that were made of hardened steel and disposed on a holder (10 mm in diameter) at 120° to each other. The load was applied with an AutoGraph AG-X (Shimadzu Corp., Kyoto, Japan) universal testing machine by means of a plain head of 1.6 mm in diameter at a speed of 1 mm·min^−1^ up the failure. The specimen thickness was measured at the breakage point. The strength value was calculated by averaging the data after testing 12 sintered disks. The equation for strength calculations was described in a previous work [28].

### 2.7. Measurement of Electrical Resistance

Electrical conductivity was determined using an RLC-78105G high-frequency meter (Good Will Instrument Co., Ltd., New Taipei City, Taiwan) in the frequency range from 20 Hz to 5 MHz. To ensure identical electrical contact between the samples and the device electrodes, silver-based conductive adhesive Rexant Kontaktol-Avto (SDS-Group, Putilkovo, Russia) with a specific electrical resistance of 0.0 Ohm·mm^2^ was applied on the polished surfaces.

## 3. Results and Discussion

**Figure 1:**
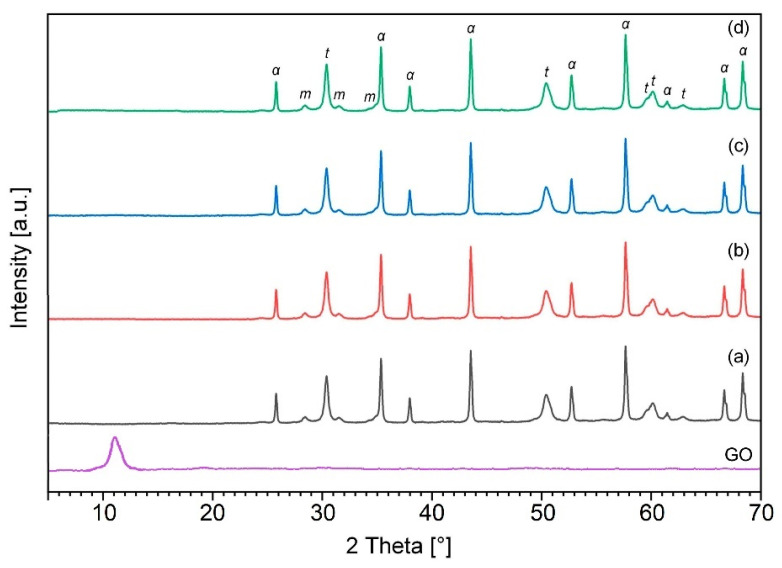
XRD analysis of fabricated GO and ZTA-GO powders after wet ball-milling containing 0 wt.% GO (a); 0.28 wt.% GO (b); 0.52 wt.% GO (c) and 6.1 wt.% GO (d). “α”, “*t*” and “m” denote corundum, tetragonal and monoclinic ZrO_2_, respectively.

**Figure 2:**
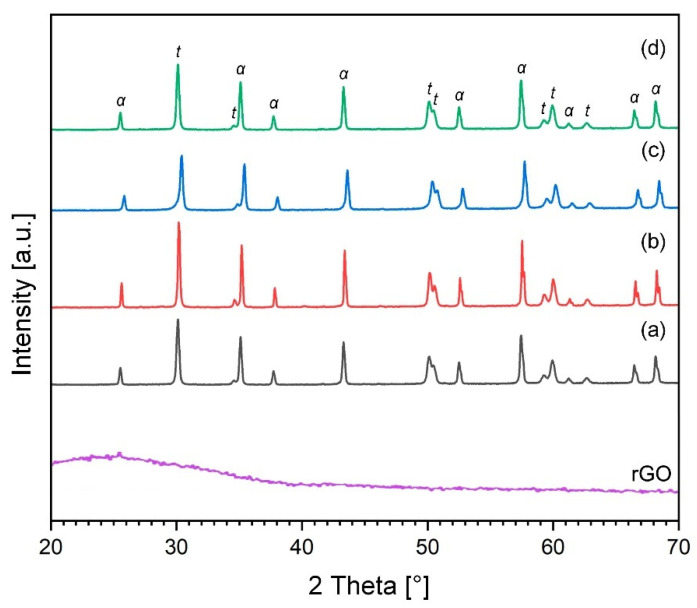
XRD analysis of rGO and sintered ZTA-GO composites containing 0 wt.% GO (a); 0.28 wt.% GO (b); 0.52 wt.% GO (c) and 6.1 wt.% GO (d). “α” and “*t*” denote corundum and tetragonal ZrO_2_, respectively.

Representative X-ray diffraction patterns corresponding to powder mixtures and sintered composites are shown in Figure 1 and Figure 2, respectively. The patterns show that no contaminants or new phases were detected both during the preparation of the mixtures and after the sintering of the powder.

X-ray diffraction analysis of the initial powder mixtures demonstrated a small broadening of the peaks (Figure 1). It is known that the typical (002) peak of graphite, which is located at *2θ* = 26.5° for the fabricated graphene oxide, moved to its most intense peak located at *2θ* ~ 11.2°. Graphite is not presented in the GO curve, which confirms its complete oxidation due to the formation of various oxygen functional groups. A well-defined peak (002) centered at ~25° on the diffractogram of rGO is observed after the thermal reduction of graphene oxide during sintering (Figure 2). Compared with graphene oxide, the shift of the peak to a higher *2θ* value can be explained by the removal of functional groups. On the other hand, after completion of the sintering process, the presence of sharp diffraction peaks was observed, which confirms the crystallinity of obtained ceramic samples (Figure 2). However, the diffraction patterns of both raw and sintered powder mixtures do not show any trace of graphene oxide because of the low volume level of the graphitic structures, which could be below the detection level by this method.

In addition, XRD analysis of the samples showed complete conversion of the monoclinic ZrO_2_ to tetragonal ZrO_2_ after SPS, as shown in Figure 2.

**Figure 3:**
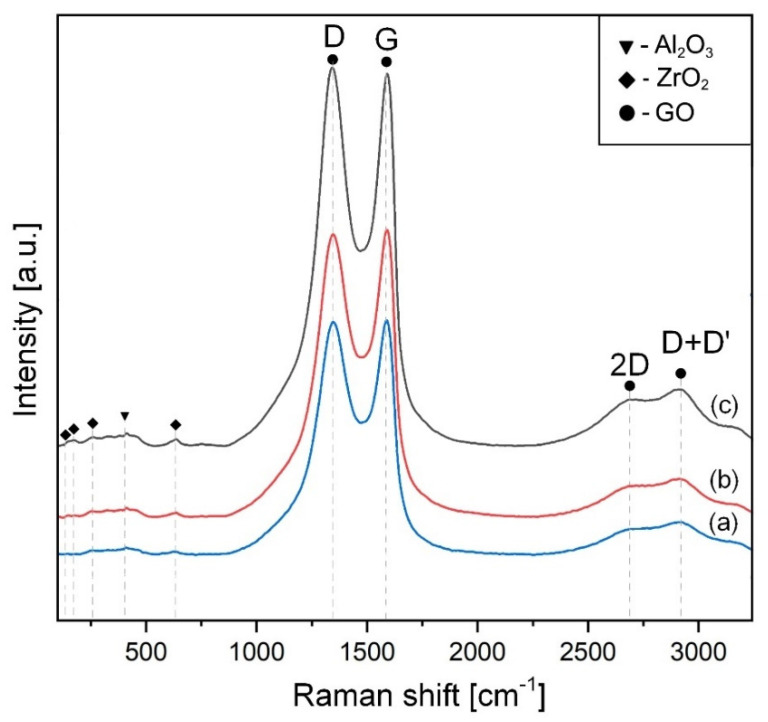
Raman spectra of as-prepared ZTA-GO mixtures containing 0 wt.% GO (a); 0.28 wt.% GO (b); 0.52 wt.% GO (c). “D”, “G”, “2D” and “D + D′” denote GO peaks.

Raman spectroscopy is a powerful tool to analyze the possible thermal reduction of GO along the entire volume of the composite. The Raman spectra of the initial powder mixtures are presented in Figure 3.

**Figure 4:**
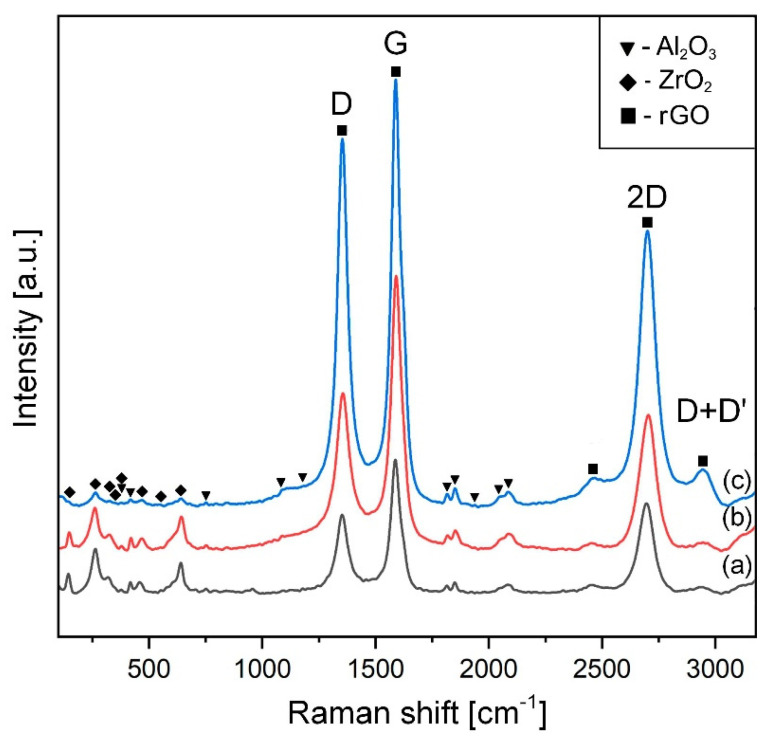
Raman spectra of sintered composites containing 0 wt.% GO (a); 0.28 wt.% GO (b); 0.52 wt.% GO (c).

The wide G peak and slight second-order area are features of sp^1^, sp^2^, and sp^3^ hybridized C–C bonds in graphene [39] and the D band (∼1350 cm^−1^) shows structural flaws, such as the lattice deformation [41]. The reduced intensity of the peaks in graphene oxide is associated with the decrease in the amount of GO. Figure 4 illustrates Raman spectra for the SPSed materials. 

These spectra indicate that GO was in situ reduced (rGO) during SPS. This is confirmed by the decrease in the intensity ratio between the D- and G-bands (I_D_/I_G_) It was found that this intensity in the raw powder mixtures reached 1.01, 0.98, and 0.99 for 0.5, 1, and 5 vol.% of GO, respectively. On the other hand, in the sintered composites, I_D_/I_G_ diminished to 0.38 and 0.51 for 0.28 and 0.52 wt.% rGO, respectively, which proves less defectivity of the rGOs into the thermally treated samples. As for the SPSed composites with 6.1 wt.% rGO, this ratio was 0.85, highlighting the presence of many defects. Perhaps thermal reduction of the composition with a high content of graphene oxide requires more dwelling time at the maximum temperature. However, an increase in sintering time may promote grain growth of the ceramic matrix and, consequently, deterioration of the mechanical properties. In addition, a well-resolved two-dimensional symmetric peak at ∼2700 cm^−1^ appears. An increase in the I_2D_/I_G_ ratio to 0.66 compared to the raw powder mixtures (~0.12) was found in SPSed composites, regardless of the composition. It also confirms the restoration of the graphene structure after sintering. The results confirmed the thermal reduction (including the reduction of large sp^2^ regions) of the graphene oxide during sintering at 1500 °C. 

**Figure 5:**
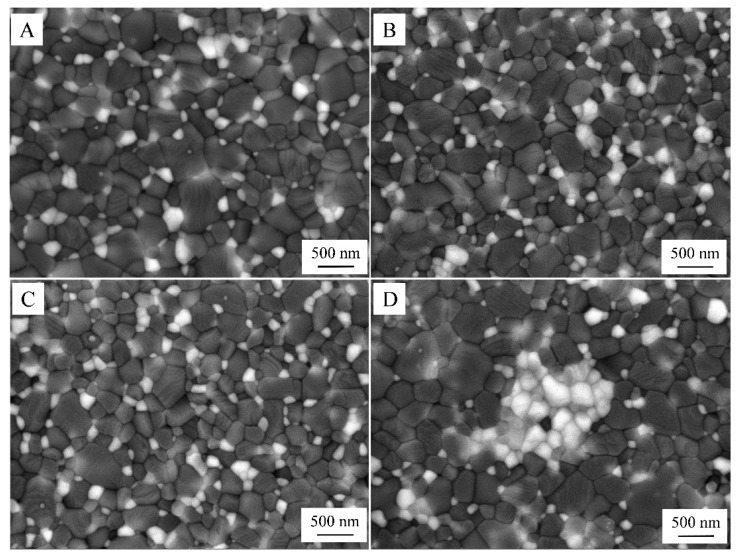
SEM images of polished surface of 0-G (**A**), 0.5-G (**B**), 1-G (**C**), and 5-G **(D**) sintered composites.

Representative electron microscope images of the microstructure of sintered samples are presented in Figure 5. In this micrograph, the dark and white phases represent Al_2_O_3_ and ZrO_2_ grains, respectively. ZrO_2_ particles were uniformly dispersed in the alumina matrix, except for the 5-G composites. The linear intercept technique was used to measure the average matrix grain size [42]. According to measurements, the following values of 0.41, 0.37, and 0.33 µm for the 0-G, 0.5-G, and 1-G composites were obtained.

Obviously, the presence of GO leads to a decrease in grain size. As for the 5-G composition, the high content of graphene oxide in the raw mixture did not allow a homogeneous distribution of ZrO_2_ and therefore the matrix grain size was the same as in the composition without graphene oxide. However, it should be noted that the average matrix particle size for all studied composites was similar (0.37 µm).

**Figure 6:**
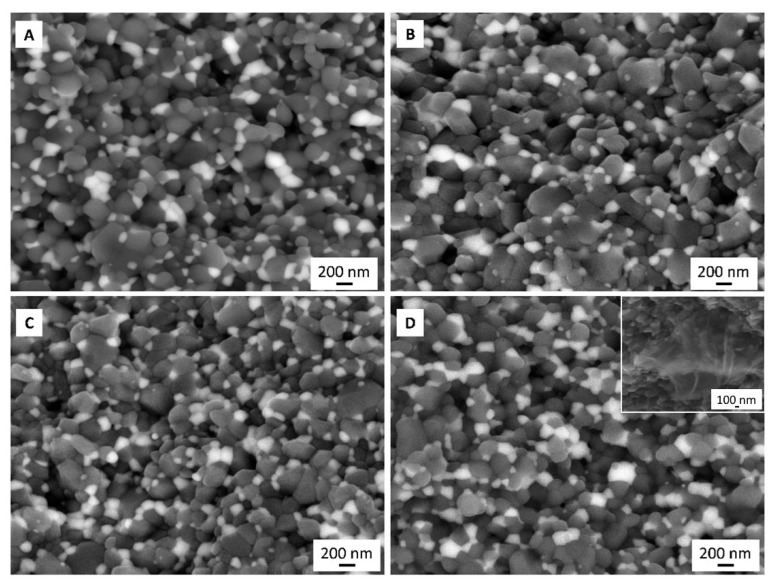
SEM images of fracture surfaces for 0-G (**A**), 0.5-G (**B**), 1-G (**C**), and 5-G (**D**) sintered composites. Close-up of fracture surface with the presence of rGO in microstructure (**D**).

The fracture surfaces of the specimens broken in the flexural loading test are shown in Figure 6.

These images confirm the uniform distribution of ZrO_2_ in the ceramic matrix. Only in the 5-G composition were agglomerates of ZrO_2_ observed. The micrograph shows that the ZrO_2_ grains are embedded around the Al_2_O_3_ grain. 

The dependence of the Vickers hardness (HV), fracture toughness (Kc), and flexural strength (σf) of the studied composites on the percentage of graphene oxide in the initial mixture is presented in Table 2. It can be stated that the mechanical performance of the composite with 0.28 wt.% GO was improved and reached values of 876 ± 43 MPa and 6.8 ± 0.3 MPa⋅m^1/2^ for strength and fracture toughness, respectively. 

Suarez et al. reported that the mechanical behavior of alumina can be improved by the presence of a small amount of graphene [22]. The clear R-curve behavior of alumina is explained by the fact that due to the low content of graphene there is a weak grain boundary, which in turn facilitates the extrinsic reinforcement mechanisms in the wake region. With increasing alumina grain size, these mechanisms become more active. In addition, the inhibiting effect of graphene on grain growth during sintering was also observed. It should also be noted that the presence of zirconium dioxide already plays the role of alumina grain growth inhibitor. The presence of GO has an insignificant effect on the Vickers hardness of the composites. The decrease in hardness in the 5-G composites is explained by the formation of pores and ZrO_2_ agglomerates at a higher rGO content in the composite matrix.

**Figure 7:**
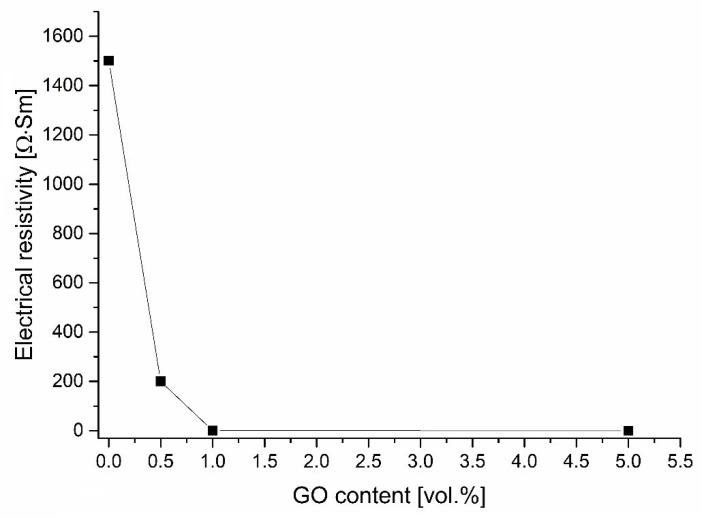
The change in electrical resistivity of sintered composites depends on the content of reduced graphene oxide.

Figure 7 demonstrates the variation of the electrical resistivity when increasing the rGO content. Pure ceramic samples show extremely high resistivity. However, from the 0.5-G composites, the resistivity is greatly reduced (below 300 Ω⋅cm^−1^), which indicates that these samples are suitable for electrical discharge machining. This proves that an addition of 0.5% rGO is already sufficient to form conductive pathways in the ceramic matrix. As the rGO content continues to increase, the electrical resistance of the samples continues to decrease.

This can be explained by the fact that with increasing rGO content there is an increase in inter-sheet bonds, which cause an improvement in conductivity along the planes a–b (orientation: perpendicular to the direction of pressure applied during sintering of the spark plasma). A serious drawback of using carbon nanotubes to fabricate composites is that they tend to form agglomerates and which inhibits contact between points. Such heterogeneity leads to a decrease in the electrical conductivity behavior of the composites. At the same time, the presence of rGO in the material leads to the emergence of contacts between lamellated graphene particles such as “area to area”. As a consequence, tightly adjacent and interconnected highly conductive carbon networks lead to lower electrical resistivity. In addition, the removal of oxygen-containing groups during thermal reduction of GO increases its specific surface area, which leads to high electron mobility and the formation of conductive pathways resulting in improved electrical conductivity.

**Figure 8:**
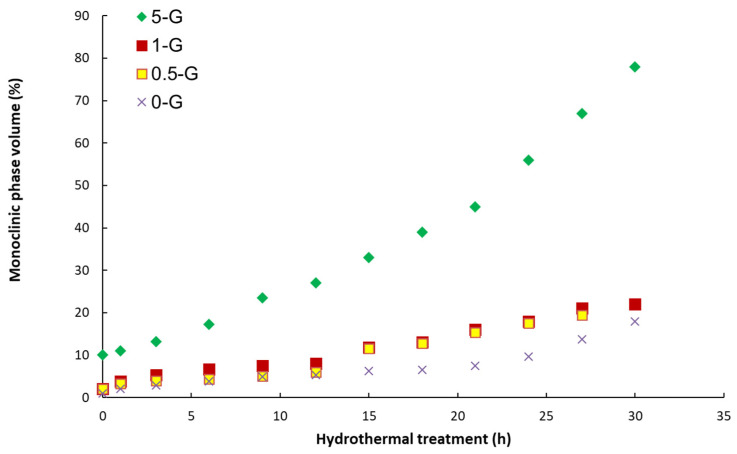
LTD—evolution of the volume fraction of monoclinic transformed ZTA-G in dependence on the aging treatment time.

Figure 8 shows the results of the aging experiments.

At the beginning of the low-temperature degradation process, the monocline phase content on the polished surfaces was 1%, 2%, 4%, and 10% for composites with 0, 0.28, 0.52, and 6.1 wt.%. GO, respectively. The significant difference between these values appears after 10 h in the autoclave at 134 °C. For composites with 6.1%, the amount of monoclinal phase is already 27%, which is almost three times higher than at the beginning of the experiment. While in other composites, this difference is not significant and is 5%, 6%, and 8% for 0, 0.28, and 0.52 wt.%. After 30 h in the autoclave at 134 °C, the composite with the highest content of the graphene oxide monoclinal phase is already 78%, which is more than half of the tetragonal phase that has been transformed. Whereas only 18%, 20%, and 22% of the initial t-ZrO_2_ transformed into m-ZrO_2_ in the composites with 0, 0.28, and 0.52 wt.% GO, respectively. The resulting monoclinal phase values in the newly developed composites are below the ISO 13356 limit of 25% for yttrium-stabilized tetragonal ZrO_2_-based ceramics [43]. Therefore, these materials may be promising candidates for hard tissue replacement applications. Of particular note is the optimal compromise between mechanical properties and aging resistance that the 0.5-G composite demonstrated in terms of long-life expectancy due to the homogeneous distribution of rGO and ZrO_2_ in the Al_2_O_3_ matrix.

## 4. Conclusions

ZTA-GO composites (containing 0.28, 0.52, and 6.1 wt.%. GO) were manufactured through a wet processing route and spark plasma sintering. The use of low GO content as an additive to the ZTA ceramic matrix enhances fracture toughness and hardness at the same time. The highest strength (876 MPa), fracture toughness (6.8 MPa·m^1/2^), and hardness (17.6 GPa) were obtained for the composites with the lowest GO content. Additionally, these composites also demonstrated increased resistance to low-temperature degradation. Thanks to their combination of mechanical performance and phase stability, we feel that this composition is a suitable alternative for medical implants. Moreover, the presence of carbon structures leads to sufficient conductivity of the studied composites for electrical discharge machining. The compositions with graphene oxide of more than 0.28 wt.% demonstrate the loss of mechanical properties by the agglomerates of the graphene structures.

## Figures and Tables

**Table 1:** Properties of Al_2_O_3_ and yttrium-stabilized tetragonal ZrO_2_ (Y-TZP) ceramics.

Property/Characteristic	Alumina [4]	Zirconia [5]
Composition	Al_2_O_3_	Y–TZP
Flexural strength	400–580 MPa	700–1500 MPa
Vickers hardness	18.0–23.0 GPa	11.0–12.5 GPa
Fracture toughness	3.3–4.2 MPa·m^1/2^	4.5–20 MPa·m^1/2^
Elastic modulus	380 GPa	210–233 GPa
Mean grain size	<1.8 µm	0.1–0.6 µm
Thermal expansion coefficient	8 × 10^−6^/°C	11 × 10^−6^/°C
Density	3.98 g/cm^3^	6.00–6.05 g/cm^3^

**Table 2:** Mechanical properties and density of Al_2_O_3_-ZrO_2_/rGO composites.

Sample	Density (%ρ_th_)	Vickers Hardness HV (GPa)	Fracture Toughness *K_c_* (MPa∙m^1/2^)	Flexural Strength *σ_f_* (MPa)
0-G	99.9	16.8 ± 0.2	5.2 ± 0.3	847 ± 30
0.5-G	99.8	17.6 ± 0.3	6.8 ± 0.3	876 ± 43
1-G	99.4	17.2 ± 0.3	5.3 ± 0.1	832 ± 27
5-G	99.1	16.1 ± 0.1	5.2 ± 0.2	786 ± 21

## Data Availability

Not applicable.
